# Nd:YAG infrared laser as a viable alternative to excimer laser: YBCO case study

**DOI:** 10.1038/s41598-023-30887-3

**Published:** 2023-03-08

**Authors:** Sandeep Kumar Chaluvadi, Shyni Punathum Chalil, Federico Mazzola, Simone Dolabella, Piu Rajak, Marcello Ferrara, Regina Ciancio, Jun Fujii, Giancarlo Panaccione, Giorgio Rossi, Pasquale Orgiani

**Affiliations:** 1 CNR-IOM Istituto Officina dei Materiali, TASC Laboratory, Area Science Park, s.s.14 km 163.5, 34149 Trieste, Italy; 2 International Centre for Theoretical Physics (ICTP), Strada Costiera 11, 34151 Trieste, Italy; 3 AREA Science Park, Padriciano 99, 34139 Trieste, Italy; 4 Department of Physics, University of Milano, Via Celoria 16, 20133 Milan, Italy; 5 Department of Molecular Sciences and Nanosystems, Ca’ Foscari University of Venice, 30172 Venice, Italy

**Keywords:** Condensed-matter physics, Materials for devices, Nanoscale materials, Techniques and instrumentation

## Abstract

We report on the growth and characterization of epitaxial YBa$_{2}$Cu$_{3}$O$_{7-\delta }$ (YBCO) complex oxide thin films and related heterostructures exclusively by Pulsed Laser Deposition (PLD) and using first harmonic Nd:Y$_{3}$Al$_{5}$O$_{12}$ (Nd:YAG) pulsed laser source ($\lambda$ = 1064  nm). High-quality epitaxial YBCO thin film heterostructures display superconducting properties with transition temperature $\sim$ 80 K. Compared with the excimer lasers, when using Nd:YAG lasers, the optimal growth conditions are achieved at a large target-to-substrate distance *d*. These results clearly demonstrate the potential use of the first harmonic Nd:YAG laser source as an alternative to the excimer lasers for the PLD thin film community. Its compactness as well as the absence of any safety issues related to poisonous gas represent a major breakthrough in the deposition of complex multi-element compounds in form of thin films.

## Introduction

Oxide perovskite thin films host innumerable properties in electronics, magnetism, and optics just by tuning/doping the cation elements as well as oxygen content^1–6^. Pulsed laser deposition (PLD) has become a state-of-art thin film growth facility in the oxide community after the successful demonstration of the stoichiometric transfer of superconducting YBa$_{2}$Cu$_{3}$O$_{7-\delta }$ (YBCO) complex oxide^7^ by KrF excimer laser of wavelength $\lambda$ = 248 nm. Since then, KrF excimer lasers have emerged as a dominant tool for the growth of very high-quality complex oxide thin films^8–11^ with applications ranging from fundamental material research to advanced semiconductor manufacturing industries for devices^12–14^. However, severe limitations arise regarding the use of excimer lasers in PLD laboratories worldwide. Excimer lasers consist largely of a mixture of noble gases (96% Ne, 3.5% of Kr/Ar) and the remaining 0.05% belongs to the halogen (i.e. F/Cl) mixture in He, present in the discharge chamber. The use of excimer lasers often raises concerns about safety issues (e.g. presence of highly poisonous gases) thus requiring expensive infrastructures to allow their use. Moreover, due to the ever-increase in demand and the scarcity of noble gas resources, the cost of KrF premix gas mixture has risen tremendously in recent past years. In this respect, to reduce their consumption, industries have also incorporated ways to recycle these gases and achieved up to 85% of the gas recycling ratio with stable laser energy output^15^. Yet, the wait time for the premix gas mixtures has severely increased in these last years thus not only hindering the smooth flow of the day-to-day activities but also tremendously increasing the maintenance costs of the lasers.

To lower the costs and long wait times incurred from the non-availability of noble gas mixtures for the excimer lasers, material scientists have started to incorporate solid-state Nd:Y$_{3}$Al$_{5}$O$_{12}$ (Nd:YAG) lasers in the PLD growth process. Nd:YAG laser uses inorganic crystals for the production of highly energetic laser radiation, therefore, ruling out any safety issues regarding the presence of poisonous gases. The fundamental frequency of the Nd:YAG laser is at 1064 nm in the infrared (IR) region of the light spectrum, but by introducing the optical harmonic crystal generators, the wavelength of the laser can be pushed to the ultraviolet (UV) region i.e., 266 nm (4th harmonics) and 213 nm (5th harmonics) mimicking the excimer’s laser wavelengths. Though the use of higher harmonic generators has allowed the successful growth of oxide thin films^16–18^, limitations such as an enormous reduction in laser output energy by using harmonic generators which are known to possibly result in an incongruent ablation of the target^19^ and inhomogeneity in the laser beam profile have made them less attractive with respect to the excimer lasers.

By taking the advantage of the recent technological advancements in the Nd:YAG lasers such as uniformity in the overall laser beam profile^20^ and high stability in laser energy, we were able to deposit high-quality stoichiometric complex oxide thin films using only its fundamental harmonics at 1064 nm. In particular, the significant improvement in Nd:YAG laser technology has made it possible to produce extremely smooth films and avoid/minimize the number of droplets on the film surface. In our prior works, we have successfully demonstrated the utilization of fundamental harmonics (1064 nm) in the growth of epitaxial simple oxides such as TiO$_{2}$^20^ and V$_{2}$O$_{3}$^21–24^. Scanning tunnel microscopy (STM) and angular-resolved photo-emission spectroscopy (ARPES) experiments have indicated the excellent thin film surface quality created by Nd:YAG laser, when compared to the same materials grown by KrF excimer laser^25–28^. In this paper, we present yet another potential application of Nd:YAG laser operating at its fundamental harmonics namely, the stoichiometric transfer of complex oxide perovskite systems such as YBCO and LaNiO$_{3}$ (LNO). The use of this kind of laser in the growth of very high-quality complex oxides represents a breakthrough in the project and realization of PLD facilities with very low maintenance costs and negligible safety issues regarding the presence of poisonous gases.

## Experimental set-up using an Nd:YAG laser

**Figure 1:**
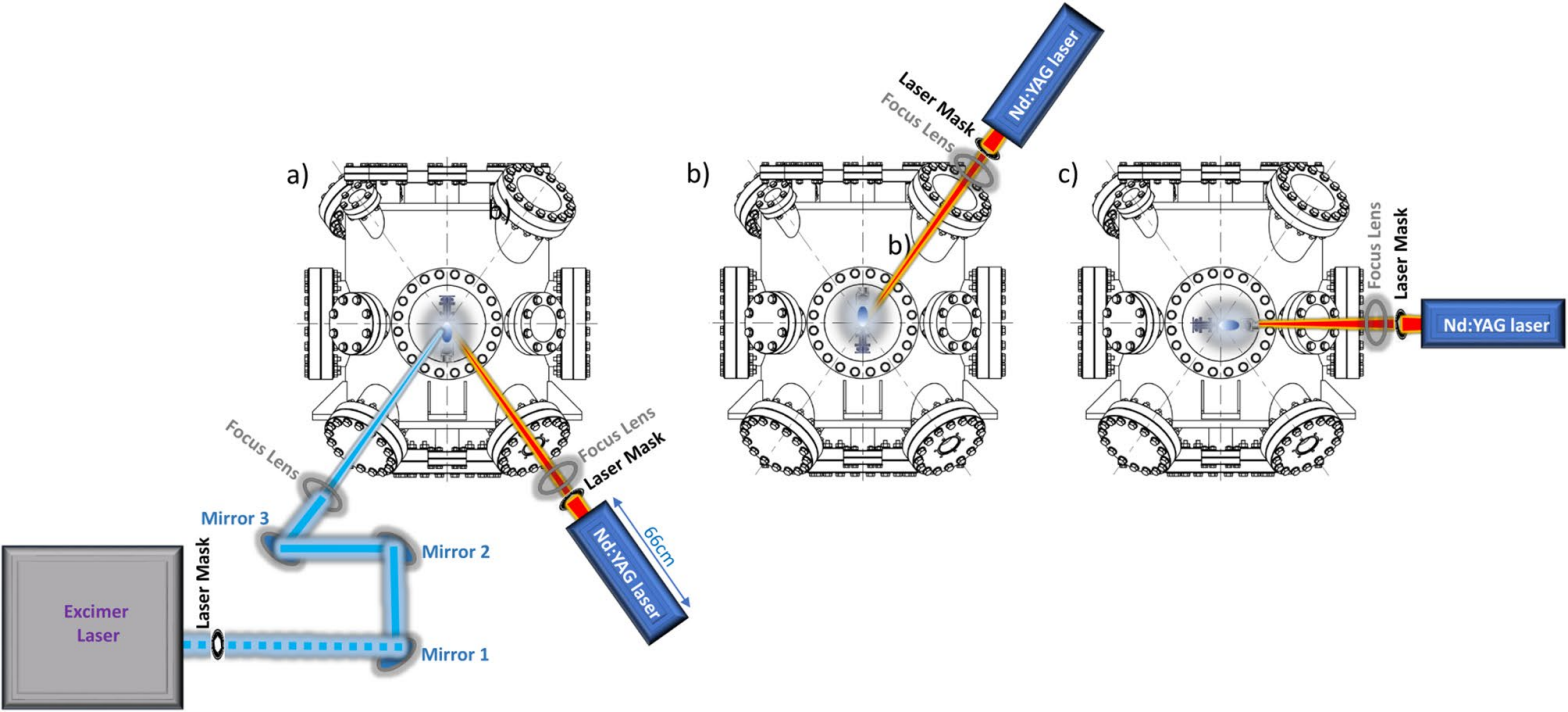
Schematic representation of our PLD system equipped with the Nd:YAG laser, laser mask, and focus lens (**a**) (typical laser-path of KrF excimer laser is also shown; other possible geometrical configurations of the laser head positioning (**b** and **c**).

The schematic of our PLD system using an Nd:YAG laser source is shown in Fig. 1. Differently from the KrF excimer laser characterized by a very large and heavy cabinet (e.g. 1182 $\times$ 375 $\times$ 793  mm and 275 kg of weight), the laser head of an Nd:YAG laser can be placed just in front of the entry-window. Such a possibility is allowed because of the sizeably smaller dimensions/weight of a typical Nd:YAG laser head. For instance, the Innolas SpitLight Compact 400’s laser head in use in our laboratory is 390 $\times$ 135 $\times$ 91  mm ((L $\times$ W $\times$ H) in dimensions for a total weight of about 10  kg.

Its positioning does not, therefore, require the use of reflecting mirrors to guide the laser pulses inside the deposition chamber thus allowing any possible geometry such as from the top or in the planar ones shown in a, b and c of Fig. 1, respectively. Moreover, the use of the full output energy of the laser is then allowed. The laser output energy is fixed at about 700 mJ with a beam diameter of about 6  mm and a maximum repetition rate of 10 Hz. Smaller repetition rates can be easily obtained by regulating the opening of the optical cavity and the actual number of laser shots per given time. It is crucial to underline the extremely high stability in the energy of the laser pulse (i.e. $< 0.7\%$ ) making the ablation rate highly stable over time as well.

In contrast to KrF excimer lasers, where the laser output energy is controllable, the lack of Nd:YAG laser’s capacity to tune the energy has been circumvented by adopting a specific process protocol for the growth/optimization of epitaxial thin films. As a matter of fact, to reduce the total fluence of the laser pulse, an alumina ceramic piece with a variable diameter hole (e.g. 1$-$2 mm or larger/smaller) is used as a laser mask. After that, a focus lens has been placed in the laser path in order to focus the laser pulses onto the targets at an incident angle of 45$^{\circ }$.

## Deposition of superconducting YBa$_{2}$Cu$_{3}$O$_{7-\delta }$ single-layer thin films

YBCO belongs to the cuprates family and is well-known for its high superconducting critical temperature T$_{c}$ at about $\sim$ 90 K. Bulk YBCO has an orthorhombic crystal structure (a = 0.384  nm, b = 0.393 nm, c = 1.182 nm, $\alpha = \beta = \gamma = 90^{\circ }$) and the space group belongs to Pmmm^29^. However, the growth of high-quality epitaxial YBCO thin films is the utmost priority for enabling their superconducting properties. Several works have been already done on the growth of very high-quality epitaxial thin films of YBCO on various substrates by PLD technique and exclusively by using KrF excimer laser. As an alternative to the use of excimer lasers, very few groups have also tried the deposition of epitaxial YBCO thin films on SrTiO$_{3}$ or MgO substrates by using Nd:YAG laser either with 3rd ($\lambda$=355 nm)^30^ or 4th ($\lambda$=266 nm)^16,31,32^ harmonics as a pulsed laser source, respectively, both falling in the UV-range of the radiation spectrum.

**Figure 2:**
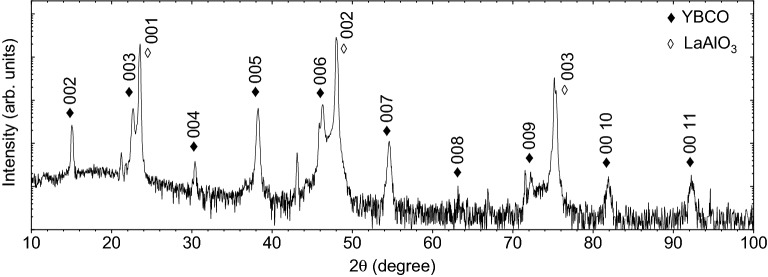
$\theta -2\theta$ XRD scan of YBCO film grown on LAO [0 0 1] substrate.

In contrast to the preceding investigations, here, we yet again used the Nd:YAG laser as a pulsed laser source operating in its fundamental harmonics (i.e., $\lambda$=1064  nm) to demonstrate the growth of epitaxial YBCO films grown on LaAlO$_{3}$ (LAO) [0 0 1] substrate. The crystallographic properties of YBCO films were checked by x-ray diffraction (XRD). In Fig. 2, the typical symmetrical $\theta -2\theta$ scan shows only the YBCO (0 0 *l*) peaks denoted as $\blacklozenge$, indicating highly textured growth along the c-axis direction. Other than the LAO substrate peaks (denoted by $\lozenge$), no further traces of impurity peaks or secondary phases can be observed.

The out-of-plane lattice parameter calculated from the (0 0 2) Bragg-reflection is $\sim$ 1.176 nm, very close to its bulk lattice constant^29^. In order to evaluate the effect of post-deposition thermal treatment, the transport properties of as-grown as well as different post-annealing conditions of YBCO films were investigated by the four-probe method.

The as-grown samples displayed very low T$_{c} \sim$ 8 K. This feature can be explained by the low oxygen deposition pressure, i.e. 5 $\times \,10^{-2}$ mbar, thus resulting in oxygen deficiencies in the YBCO as-grown films. To compensate for the oxygen deficiencies, the films were post-annealed at different annealing temperatures and oxygen pressure conditions. The films that were mildly post-annealed (500 $^{\circ }$C, 100 mbar oxygen, 60  min) show an improvement in T$_{c}$ from 8 to 62 K. Whereas, the film that underwent extreme post-annealing (600 $^{\circ }$C, 300 mbar oxygen, 60 $-$ 80  min) showed typical superconducting behavior with a linear slope, and its T$_{c}^{onset}$ increased from 55 to 93 K, indicating an increase in oxygenation in the film.

**Figure 3:**
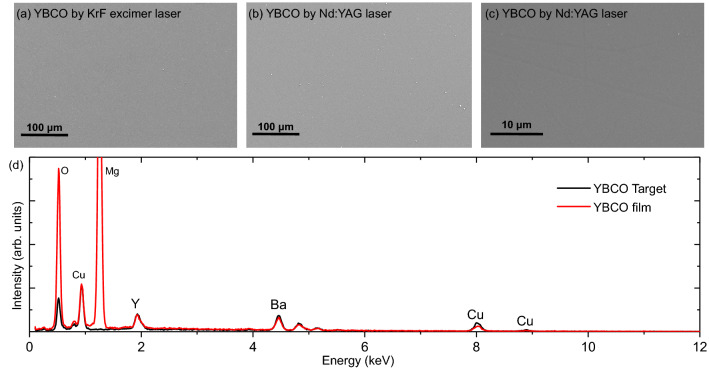
SEM images of the YBCO thin films grown using (**a**) KrF Excimer laser and (**b** and **c**) first harmonic Nd: YAG solid-state laser are presented. (**d**) Comparison of the EDS spectra of a YBCO target (black) and a YBCO film (red) grown by the first harmonic Nd: YAG laser.

To assess the microstructural surface quality of the thin films produced with the first harmonic Nd:YAG laser, a comparison of Scanning Electron Microscopy (SEM) images between the films grown using the conventional KrF Excimer laser and the new-generation Nd:YAG solid-state laser is presented in Fig. 3. Contrary to prior reports of droplets appearing in PLD-grown YBCO films^33–35^, the surface quality of the YBCO film grown with the first harmonic Nd:YAG laser, as shown in Fig. 3b and c, is smooth and free of droplets, similar to the film surface grown using the KrF laser (as depicted in Fig. 3a). The stoichiometric transfer of elements can be verified by comparing the Energy Dispersive Spectroscopy (EDS) line profiles of the YBCO target and the YBCO film, which are identical within the constraints of the instrumentation, as demonstrated in Fig. 3d. The structural, superconducting properties, microstructural and composition analysis of YBCO films reveal that the new-generation Nd:YAG lasers are equally comparable to the traditional KrF laser source.

## Deposition of functional oxides thin films

In order to further assess the congruent ablation of multi-element complex oxide materials by Nd:YAG lasers operating at their first harmonics, highly metallic LaNiO$_{3}$ (LNO) and insulating CeO$_{2}$ were also deposited on LAO substrate. Because of its metallic character, LNO is one of the most technologically important materials and is frequently employed as a counter electrode in devices^36–38^. Differently, the insulating CeO$_{2}$, besides being extensively investigated for many environmental-friendly applications such as solid oxide fuel cells, water splitting for hydrogen production, and oxygen sensors^39–41^, has been widely used as a function buffer-layer for the YBCO growth in form of thin films^42^.

**Figure 4:**
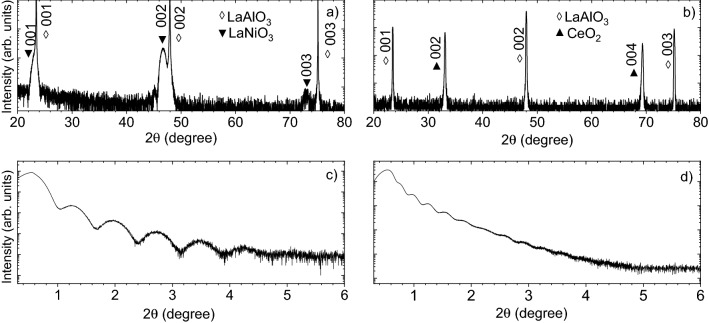
$\theta - 2\theta$ out-of-plane X-Ray Diffraction of the LNO (**a**) and CeO$_{2}$ (**b**) films grown on LAO [0 0 1] substrate, showing only (0 0 *l*) diffraction peaks; typical low-angle x-ray reflectivity curves of LNO (**c**) and CeO$_{2}$ (**d**) film displaying clear oscillations up to $2\theta$ of 4$^{\circ }$–5$^{\circ }$.

LNO has trigonal R-3c space group with lattice constant a = 0.54535 nm and c = 1.31014  nm^43^, but along the [012]-direction, a nearly cubic perovskite structure with a lattice constant of 0.383 nm can be identified, making it easily adaptable to most perovskite systems. The bulk structure of CeO$_{2}$ is a cubic fluorite type with lattice parameter of a = 0.54097  nm^44^. When CeO$_{2}$ is grown on LAO, the in-plane lattice cell results rotated 45$^{\circ }$ with an in-plane lattice parameter of about 0.38252  nm, thus very close to those of the substrate (i.e. 0.379 nm). Therefore, both these materials are promising candidates in growing multi-functional heterostructures hosting a superconducting YBCO layer. From the wide angular $\theta -2\theta$ XRD scan (panels a and b in Fig. 4), both the LNO and the CeO$_{2}$ films display only a single crystalline phase of (0 0 *l*) reflections without any indications of secondary impurity phases.

The calculated out-of-plane lattice constant of LNO film is $\sim$ 0.391 nm which is larger than the bulk lattice constant, thus confirming the compressive strain imposed by the LAO substrate. On the contrary, the out-of-plane lattice constant of CeO$_{2}$ film is $\sim$ 0.541 nm, thus inferring the substantial relaxation of the films and the absence of any substrate-induced strain mechanisms imposed by the substrate.

Film thickness and its surface roughness were then investigated by low-angle x-ray reflectivity (XRR), as shown in panels (c) and (d) in Fig. 4. Simulations of the low-angle XRR were performed by means of the IMD package of XOP software^45,46^. Besides the evaluation of the thickness of grown layers, XRR curves provide a direct estimate of the surface roughness being their intensity dumped for its higher and higher value. In the case of LNO, the XRR oscillations can be seen for 2$\theta$ values up to 5$^{\circ }$ and above it, the oscillations fall below the experimental sensitivity of the x-ray diffractometer. Similarly, in the case of CeO$_{2}$ film, XRR oscillations were recorded up to 2$\theta$ values of about 4.5$^{\circ }$, and, similarly to the LNO case, above this angle, the oscillations fell below the experimental sensitivity of the x-ray diffractometer. Even though the XRR fitting algorithm is based on a monochromatized X-ray source with negligible lateral inhomogeneities of the beam (while we used a lab-based unmonochromatized X-ray beam), the surface root-mean-square (RMS) roughness was therefore estimated to be $\sim$0.4 nm, corresponding to about one single LNO/CeO$_{2}$ unit cell and therefore inferring a very low surface roughness for both the functional layers.

**Figure 5:**
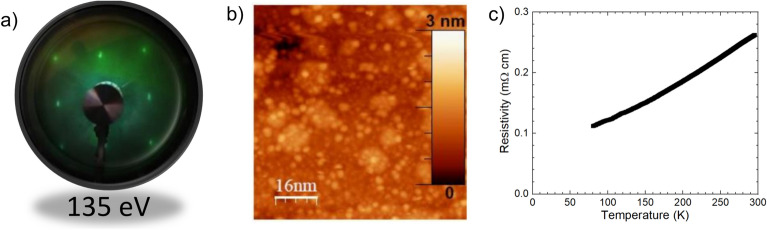
(**a**) LEED of an LNO film taken at 135 eV displays sharp diffraction spots with square lattice, (**b**) STM topography of the film, and (**c**) its typical temperature-dependent resistivity.

Being LNO metallic at room temperature, a more detailed characterization of the structural and electronic properties of LNO can be obtained by Low-Energy Electron Diffraction (LEED) and Scanning Tunnel Microscopy (STM). Figure 5a shows LEED with very sharp diffraction spots indicating the square lattice plane without any secondary phase present in the films. In addition, the in-situ STM topography analysis on LNO (Fig. 5b) shows a very flat film surface with the RMS surface roughness of roughly $\sim$ 0.3 nm i.e., equivalent to less than one unit cell roughness. Finally, temperature-dependent electrical transport measurement on the LNO film (Fig. 5c) shows metallic behavior down to 77 K and the resistivity measured at 300 K is $\sim$ 0.26 m$\Omega$ cm, which is comparable with the films grown by KrF lasers.

## Deposition of oxide multilayers

**Figure 6:**
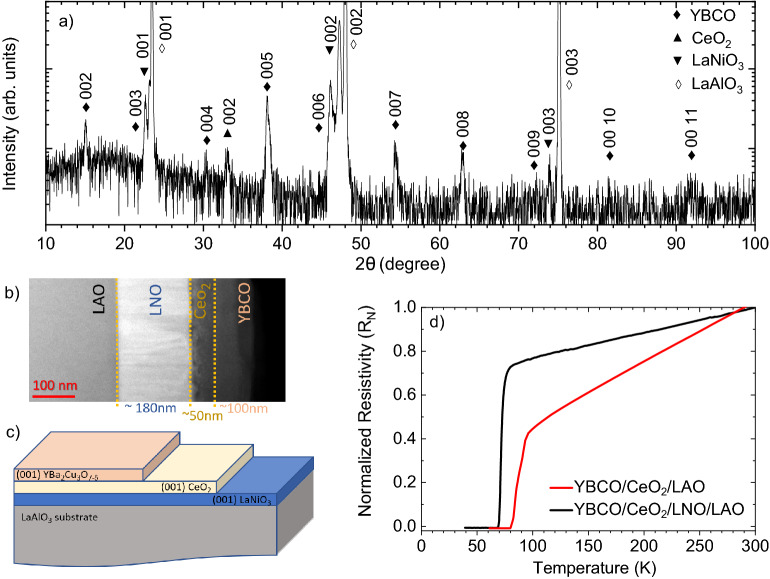
(**a**) $\theta -2\theta$ scan of the YBCO/CeO$_{2}$/LNO heterostructure grown on LAO [0 0 1] substrate, (**b**) a cross-sectional HAADF-STEM image and (**c**) a schematic representation of the YBCO/CeO$_{2}$/LNO/LAO heterostructure stack; (**d**) room-temperature normalized resistance vs temperature behavior of the YBCO/CeO$_{2}$ and YBCO/CeO$_{2}$/LNO stack displaying T$_{c}^{zero} \sim$ 80 K and 70 K respectively.

Till now, we have illustrated the epitaxial growth of individual oxide layers. To further highlight the adaptability and development of the Nd:YAG pulsed laser source, a heterostructure stack (Figure 6(c)) made up of layers of YBCO, CeO$_{2}$, and LNO has deposited on LAO [0 0 1] substrate. The growth of each layer was carried out under optimal conditions. All the layers were deposited at the same substrate temperature i.e., 720$^{\circ }$C while the oxygen background pressure for the growth of each layer was different. In particular, LNO, CeO$_{2}$, and YBCO were grown at 10$^{-1}$, 5$\times \,\,10^{-4}$, and 5$\times \,\,10^{-2}$ mbar, respectively. In order to oxygenate the YBCO functional layer, the samples were post-annealed for about 60–80 min at 600 $^{\circ }$C in an oxygen atmosphere at 300 mbar.

The XRD was used to assess the structural quality of the heterostructure stack deposited on LAO substrate. As expected, only (0 0 *l*) oriented peaks of the YBCO ($\blacklozenge$), CeO$_{2}$ ($\blacktriangle$) and LNO ($\blacktriangledown$) layers were seen in Fig. 6a, confirming the preferential orientation along the [0 0 1] crystallographic direction for all of the layers. Moreover, the absence of any other diffraction peaks besides those related to the three layers ruled out the presence of any spurious phases.

High-Angle Annular Dark Field (HAADF) Scanning Transmission Electron Microscopy (STEM) was used to investigate the nanostructure of the film. In Fig.  6b, HAADF-STEM cross-sectional image of the film highlights abrupt, high-quality, epitaxial interfaces with the underlying substrate and among the different oxide layers. The structure of the film over the whole image is homogeneous and free of significant defects. In particular, no evidence of the segregation of droplets was observed. The measured thickness of the oxide layers are YBCO of 100 nm, CeO$_{2}$ of 50 nm and LNO of 170 nm. Figure 6d shows the temperature-dependence resistance of both YBCO/CeO$_{2}$/LAO and YBCO/CeO$_{2}$/LNO/LAO stack, respectively. The resistance shows a linear dependence of the resistivity from room temperature down to superconducting transition temperature with no sign of either up(down)ward curvature as typical for nearly-optimally doped samples^47,48^. The YBCO layer grown on the CeO$_{2}$ buffered LAO substrate demonstrated superconductivity at approximately 80 K, while the T_c_ of the CeO$_{2}$/LNO/LAO stack was slightly decreased to 70 K. This decrease in T_c_ can be attributed to structural defects in the tri-layer structure. Nevertheless, in both cases, the superconducting phase of the YBCO over-layer shows a sharp transition to zero resistance.

## Discussion and conclusions

We have successfully established the growth protocols of epitaxial complex oxide thin films by using the first harmonic Nd:YAG laser operating at wavelength $\lambda$ = 1064 nm. Thanks to the new-generation solid-state laser qualities such as stability, homogeneity, and longevity of the lasers, good quality oxide films with high reproducibility are achieved. The overall maintenance cost is tremendously reduced as neither the optical harmonic generators nor the excimer lasers noble/poisonous gas mixture is required, which is even more environmentally friendly. As a result, the first harmonic Nd:YAG solid-state lasers are undoubtedly a powerful substitute for traditional excimer lasers for the deposition of oxide thin films.

## Methods

The Nd:YAG fundamental harmonics ($\lambda$ = 1064 nm) was used as a pulsed laser source for the deposition of ternary complex oxide systems. The pristine spot size of the laser shot is about 6 mm in diameter with a typical energy of 700 mJ, corresponding to an energy density of about 2.5 J cm$^{-2}$ for the unfocused beam. With the dual aim of avoiding the peripheral region of the laser spots as well as reducing the growth rate per laser shot, an optical mask was used to reduce the spot size from 6 to 2 mm in diameter.

Epitaxial thin films of YBCO, LNO and CeO$_{2}$ were deposited on LAO [0 0 1] substrate by Nd:YAG solid-state laser operating at its first harmonics. All of the films were deposited at 720 $^{\circ }$C substrate temperature and using a laser repetition rate of 1 Hz. The typical deposition rate was about 0.35 nm min$^{-1}$. Following deposition, the YBCO thin films were post-annealed at various annealing temperatures (500 $^{\circ }$C and 600 $^{\circ }$C) and oxygen pressures (100 mbar and 300 mbar) for about 60–80 min. Compared to the films deposited by the KrF Excimer lasers where the typical target-to-substrate distance *d* is maintained at 4–5 cm, optimal growth conditions were obtained with a *d* value of about 8–10 cm for the films deposited by the Nd:YAG laser.

The crystalline structure, thickness, and surface roughness of films were probed by a four-circle Panalytical X’pert diffractometer with a Cu K$_{\alpha _{1}}$ radiation source. The surface morphology and the long-range crystalline ordering were investigated by the in-situ STM and LEED, respectively. A standard four-probe Van-der-Paw method was used for the investigation of temperature-dependent electrical transport properties of the films.

The surface morphology of the YBCO films was studied using a Supra 40 field-emission gun (FEG) Scanning Electron Microscope (SEM) equipped with a Gemini column and an In-lens detector, providing an improved signal-to-noise ratio. The chemical composition of the samples was analyzed through Energy Dispersive Spectroscopy (EDS) experiments using an Oxford LN2-free X-Act Silicon Drift Detector. The results were then processed using Aztec software to calculate the chemical composition of the samples.

HAADF-STEM experiments were performed using a JEOL 2010 UHR TEM equipped with a field emission gun and operated at 200 kV. Microscopy data analysis was performed with the Gatan Microscopy Suite 3.20.1314.0 (GMS). HAADF-STEM images were acquired using an illumination angle of 12 mrad and collection angle 88 $\le 2\theta \le$234 mrad. Cross-sectional TEM samples were prepared with a conventional polishing technique followed by dimpling and milling with Ar ions. This preparation procedure had been proven to minimize structural and chemical modifications of cross-sectional TEM samples and had successfully been applied to other oxide thin-film systems^49–51^.

## Data Availability

The datasets generated during and/or analysed during the current study are available from the corresponding author on reasonable request.
